# Combination of Extrusion and Drop‐on‐Demand Bioprinting in One Process Enables the Local Placement of Cells or Signaling Factors Into (Bio) Printed Hydrogel Structures

**DOI:** 10.1002/elsc.70062

**Published:** 2025-12-30

**Authors:** Finn Dani, Nieves Cubo‐Matteo, Leonie Schlicht, Michael Gelinsky, Anja Lode

**Affiliations:** ^1^ Centre for Translational Bone Joint and Soft Tissue Research Faculty of Medicine Technische Universität Dresden Dresden Germany; ^2^ Grupo de Investigación ARIES, Universidad Nebrija Madrid Spain

**Keywords:** bioprinting, cell migration, cell placement, drop‐on‐demand, organoid

## Abstract

Combining the volumetric fabrication of hydrogel constructs using extrusion bioprinting with highly precise drop‐on‐demand (DoD) bioprinting offers exciting opportunities in biofabrication. This technical report presents a technique in which a solenoid micro‐pipette is operated as an additional tool in an extrusion (bio)printing system to deposit small volumes of bioinks into extrusion‐printed hydrogel constructs. Using three exemplary approaches, we show that this enables the patterned placement of cells or growth factors within 3D constructs and thus influences developmental processes. Human cells within low‐viscosity bioinks, deposited into extrusion‐printed hydrogel constructs by filling inter‐strand cavities or by injection into the hydrogel strands, maintained their viability and functionality up to 28 days. As demonstrated for salivary gland cells, the properties of the hydrogel matrix can influence the fate of the injected cells: In a stiff alginate (Alg)‐based hydrogel, they formed aggregates, which is beneficial for organoid formation, and in softer hydrogels, they migrated to neighboring cell clusters. Locally injected signaling factors such as vascular endothelial growth factor (VEGF) attracted endothelial cells and fibroblasts, which migrated into previously cell‐free hydrogel areas. The combination of extrusion and DoD bioprinting opens new approaches to integrate different cell types and functionalizations in one construct, facilitating the creation of more complex and dynamic models.

**Abbreviations**
Alg: alginate
C: collagen type I
cLSM: confocal laser scanning microscopy
DMEM: Dulbecco's modified Eagle's medium
DoD: drop‐on‐demand
F: fibrinogen
FBS: fetal bovine serum
HCM: hypoxia‐conditioned medium
hMSGC: human minor salivary gland cells
HUVEC: human umbilical vein endothelial cells
MC: methylcellulose
NHDF: normal human dermal fibroblasts
PBS: phosphate‐buffered saline
VEGF: vascular endothelial growth factor

## 1 Introduction

Extrusion‐based 3D bioprinting, the strand‐wise deposition of cell‐loaded hydrogels, and drop‐on‐demand (DoD) bioprinting, the placement of cell‐loaded droplets, are commonly used and well‐established techniques in biofabrication. Each of them offers distinct advantages: Extrusion bioprinting excels at depositing high‐viscosity bioinks with high shape fidelity to construct robust and volumetric cell‐loaded constructs of clinically relevant size [1, 2]. DoD bioprinting allows for the precise dispensing of low‐viscosity bioinks with volumes in the nanoliter range and even of single cells [3]. The combination of both techniques would make it possible to unite volumetric 3D bioprinting with high‐precision placement of biological components, such as cells or signaling factors, to expand the possibilities for reproducing the complex architecture of native tissues. However, up to now, there are only a few studies which applied both techniques in one fabrication process of complex tissue models: A human skin model was engineered by extrusion printing a collagen‐based biomaterial ink and a polycaprolactone mesh before homogenously distributing keratinocytes on top using inkjet printing; furthermore, extrusion bioprinted adipose stem cells were combined with DoD bioprinted endothelial cells in one construct [4, 5]. Using an acoustics‐based DoD method, endothelial cells were pattered around extrusion bioprinted liver‐cell spheroids [6].

This technical report introduces a DoD system based on a micro‐pipette that can be operated by an extrusion bioprinting system (see Supporting Information). The micro‐pipette collects (bio)inks from a reservoir and deposits them on a surface or into a hydrogel construct. Thus, while the number of different (bio)inks for extrusion printing is limited by the number of channels of the printer, the combination with the micropipette allows for the addition of numerous (bio)inks. In this study, the potential of this combined printing approach was explored on the basis of three application examples.

Practical application: Integrating extrusion and DoD bioprinting into one fabrication process expands the possibilities to integrate different cell types and functionalizations into one construct in a precise manner, to direct development and interactions of integrated cells, and ultimately to create more complex and dynamic in vitro models for tissue engineering applications and fundamental research in biology and biotechnology. The technique is expected to advance the fabrication and in vitro maturation of tissue‐like constructs as well as the formation of organoids. Both applications are of high relevance in biomedical research, for example, for the creation of disease models to enhance understanding of the mechanisms, of patient‐specific cancer models to identify effective drugs, or to establish in vitro platforms for pharmaceutical drug screening. In addition, this technique could further be applied to nonmammalian cells, with potential applications in the field of engineered living materials, such as for the development of advanced biosensors.

## 2 Materials and Methods

### 2.1 Cells and Media

Human umbilical vein endothelial cells (HUVEC) and normal human dermal fibroblasts (NHDF, isolated from the dermis of juvenile foreskin) were purchased from Promocell (Heidelberg, Germany). Human minor salivary gland cells (hMSGC, consisting mainly of mesenchymal stem cells and epithelial cells [7]) were isolated, cultured, and kindly provided by Prof. Athina Bakopoulou and Prof. Dimitrios Andreadis (School of Dentistry, Aristotle University of Thessaloniki, Greece). HUVEC were expanded in endothelial cell growth medium (Promocell), and NHDF in Dulbecco's modified Eagle's medium (DMEM; Gibco, Life Technologies, Germany) supplemented with 10% fetal bovine serum (FBS; Corning, USA) and 100 U/mL penicillin+100 µg/mL streptomycin (Gibco). HUVEC and NHDF were cocultured in a 1:1 mix of their respective medium. hMSGC were cultivated in alpha‐MEM supplemented with 15% FBS, 100 µM L‐ascorbic acid phosphate (Merck Millipore, Germany), 2 mM L‐glutamine (Merck Millipore), and a mixture of 100 U/mL penicillin+100 µg/mL streptomycin+0.25 µg/mL amphotericin B (Sigma‐Aldrich, Merck); all cells were cultured at 37°C in a humidified 5% CO_2_ atmosphere. For the experiments, HUVEC were used in Passage 5, NHDF in Passage 7, and hMSGC in Passage 9.

### 2.2 Signaling Factors

Recombinant human vascular endothelial growth factor A_165_ (VEGF) was purchased from PeproTech (Hamburg, Germany); 20 ng/mL in DMEM was used in Experiment 3 (Figure 3). Hypoxia‐conditioned medium (HCM) as a signaling factor cocktail, containing the secretome of human MSC cultured under hypoxic conditions, was generated as described [8, 9]. In brief, MSC were cultured in DMEM with 10% FBS until 95% confluency. Then, the medium was exchanged against phenol‐free DMEM (Gibco) supplemented with 2% human serum (0.057 mL/cm^2^), and the cells were cultured under gentle shaking for 5 days at 1% O_2_. After collection, HCM was centrifuged, and the supernatant was dialyzed against deionized water. 4‐mL Aliquots were directly frozen at −80°C (1 × HCM) or concentrated by freeze–drying and resuspension in 80 µL DMEM (50 × HCM) before storage at −80°C.

**FIGURE 1:**
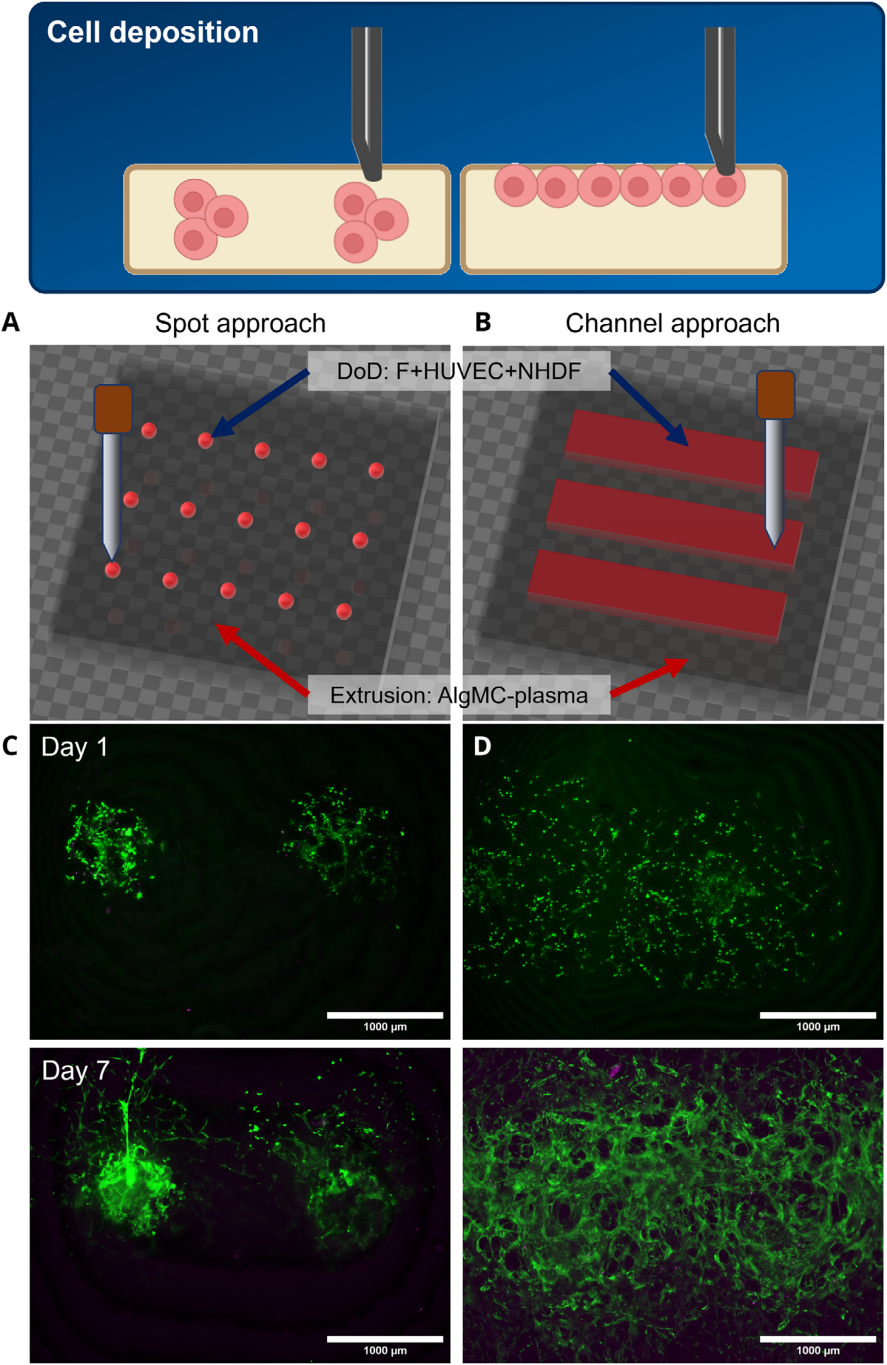
Deposition of NHDF and HUVEC cocultures by DoD printing into AlgMC–plasma hydrogel constructs. (A) Schematic representation of the printed constructs according to the “spot approach”: Cell‐free AlgMC–plasma was strand‐wise deposited as a bulk construct (12 mm × 12 mm base, 3 layers with 0.56 mm height each) by extrusion‐printing. The bioinks for DoD printing were prepared by suspending 3 × 10^6^ NHDF or 2 × 10^6^ HUVEC per mL in F ink. They were injected with the micro‐pipette in different spots 2 mm apart (five drops of F ink+NHDF per position were injected approx. 1 mm deep into the construct, and five drops of F ink+HUVEC were injected into the same position). (B) Schematic representation of the printed constructs according to the “channel approach”: Cell‐free AlgMC–plasma was strand‐wise deposited as a rectangular construct (12 mm × 12 mm base, 3 layers with 0.56 mm height each) with three channels (width: 0.84 mm) in the uppermost layer by extrusion‐printing. The channels were subsequently filled by DoD printing. The bioinks for DoD printing were prepared by suspending 3 × 10^6^ NHDF per mL in F ink or 2 × 10^6^ HUVEC per mL in culture medium. With the micro‐pipette, 25 drops of F ink+NHDF were dispensed into a channel, and after initial crosslinking for 2 min, five drops of HUVEC in medium were injected approx. 1 mm deep into the channels in positions 2 mm apart. Then, crosslinking commenced for another 8 min. (C+D) Fluorescence‐microscopic images of live (green) and dead (purple) stained cells at Days 1 and 7 of cultivation of the crosslinked constructs, visualizing 100% cell viability, cell distribution, and network formation (Day 7); three biological replicates per condition and day were analyzed; scale bar: 1000 µm.

### 2.3 Biomaterial Inks and Crosslinking Procedures

For extrusion printing, a hydrogel blend consisting of 3 wt% alginate (Alg) from brown algae (Alg; alginic acid sodium salt, Sigma) and 9 wt% methylcellulose (MC; Sigma) was used. Solvents were either deionized water (AlgMC–water) [10, 11], phosphate‐buffered saline (AlgMC–PBS) [11, 12], or fresh‐frozen human blood plasma, provided by the German Red Cross, Dresden, Germany (AlgMC–plasma) [11, 13]. In case of AlgMC–water and AlgMC–PBS, Alg was dissolved and autoclaved as solution (121°C, 20 min), autoclaved MC powder was added and the mixture was allowed to swell for 2 h. In case of AlgMC–plasma, Alg and MC were separately autoclaved as powder. On the printing day, Alg was dissolved in plasma, and after complete solution MC was added before printing immediately. For DoD printing, 15 mg/mL human fibrinogen (F; Tisseel Fibrin Sealant Kit; Baxter, USA) in PBS was used to produce the F ink; 2 mg/mL of neutralized soluble rat tail‐derived collagen type I (C; Meidrix, Esslingen, Germany) was added to obtain the CF ink. The Alg–plasma ink was prepared by dissolving 1.5 wt% autoclaved Alg powder in fresh‐frozen plasma.

**FIGURE 2:**
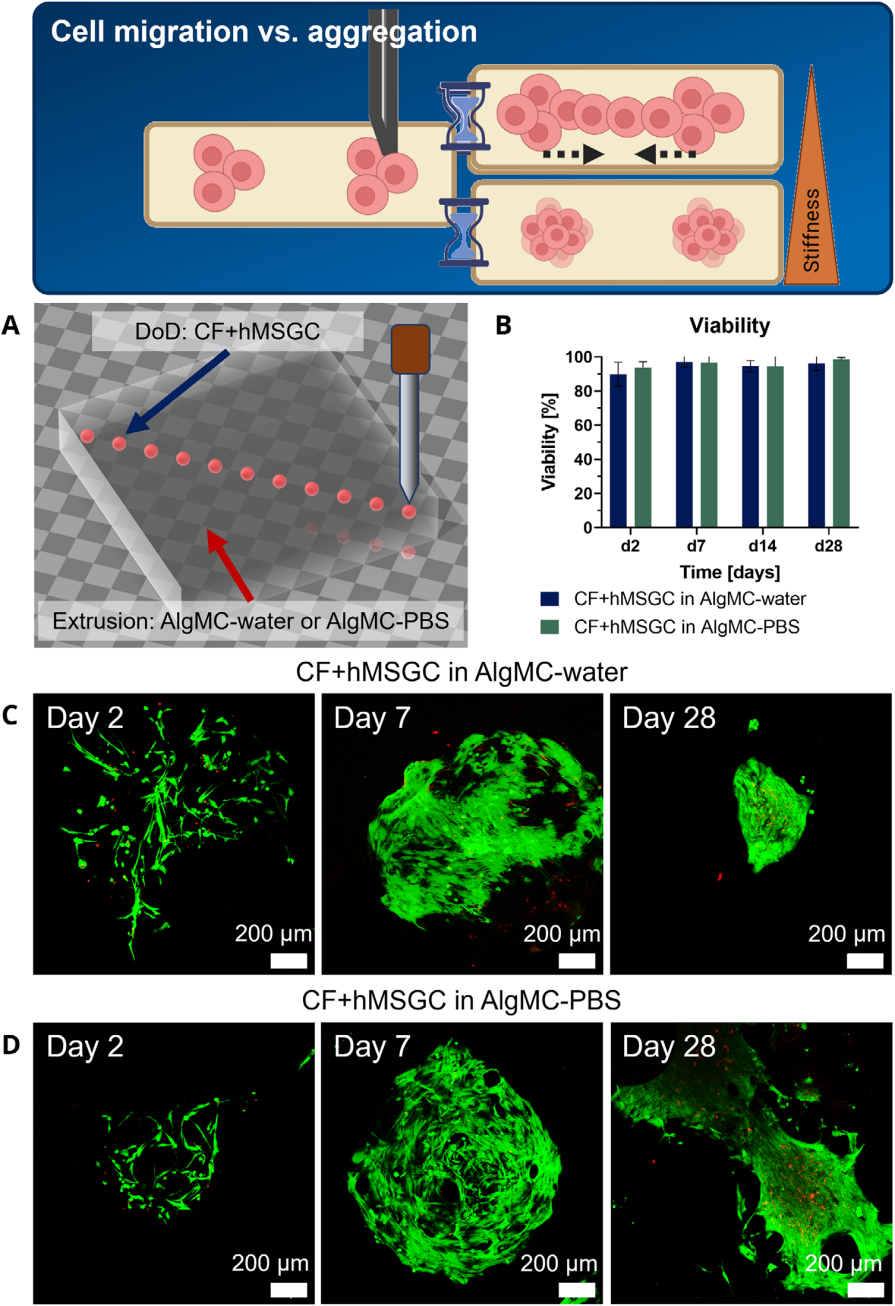
Injection of hMSGC by DoD printing into AlgMC hydrogel constructs of different stiffness. The stiffness of the surrounding hydrogel can induce either aggregation or migration of the injected cells. (A) Schematic representation of the printed constructs: Cell‐free AlgMC–water or AlgMC–PBS were strand‐wise deposited as a bulk construct (12 mm × 12 mm base, 2 layers with 0.56 mm height each) by extrusion‐printing. The hMSGC‐laden CF‐bioink, prepared by suspending 2 × 10^6^ hMSGC per mL in CF ink, was injected 1 mm deep into the constructs with the micro‐pipette at different spots (150 nL with ca. 300 cells per spot) 1.4 mm apart. (B) Viability of cells over the span of the experiment; *n* = 4. (C) The crosslinked constructs were cultivated over 28 days in hMSGC medium, and the viability was quantified based on fluorescence‐microscopic images after simultaneous staining of live and dead cells (mean±SD, *n* = 4). (D) Fluorescence‐microscopic images of live (green) and dead (red) cells, visualizing cell spreading (Day 2), proliferation (Day 7), and either aggregation or migration (Day 28); four biological samples per condition and day were analyzed; scale bar: 200 µm.

Constructs printed by combined extrusion of AlgMC inks and DoD of F‐/CF‐based bioinks (first and second experiment: Figures 1, 2) were incubated in 100 mM CaCl_2_ solution containing 0.3 iU/mL thrombin (Tisseel Fibrin Sealant Kit) for 10 min to crosslink Alg and fibrinogen. Constructs printed by combined extrusion of AlgMC (bio)inks and DoD of Alg‐plasma (third experiment: Figure 1) were crosslinked in 20 mM CaCl_2_ solution for 10 min.

**FIGURE 3:**
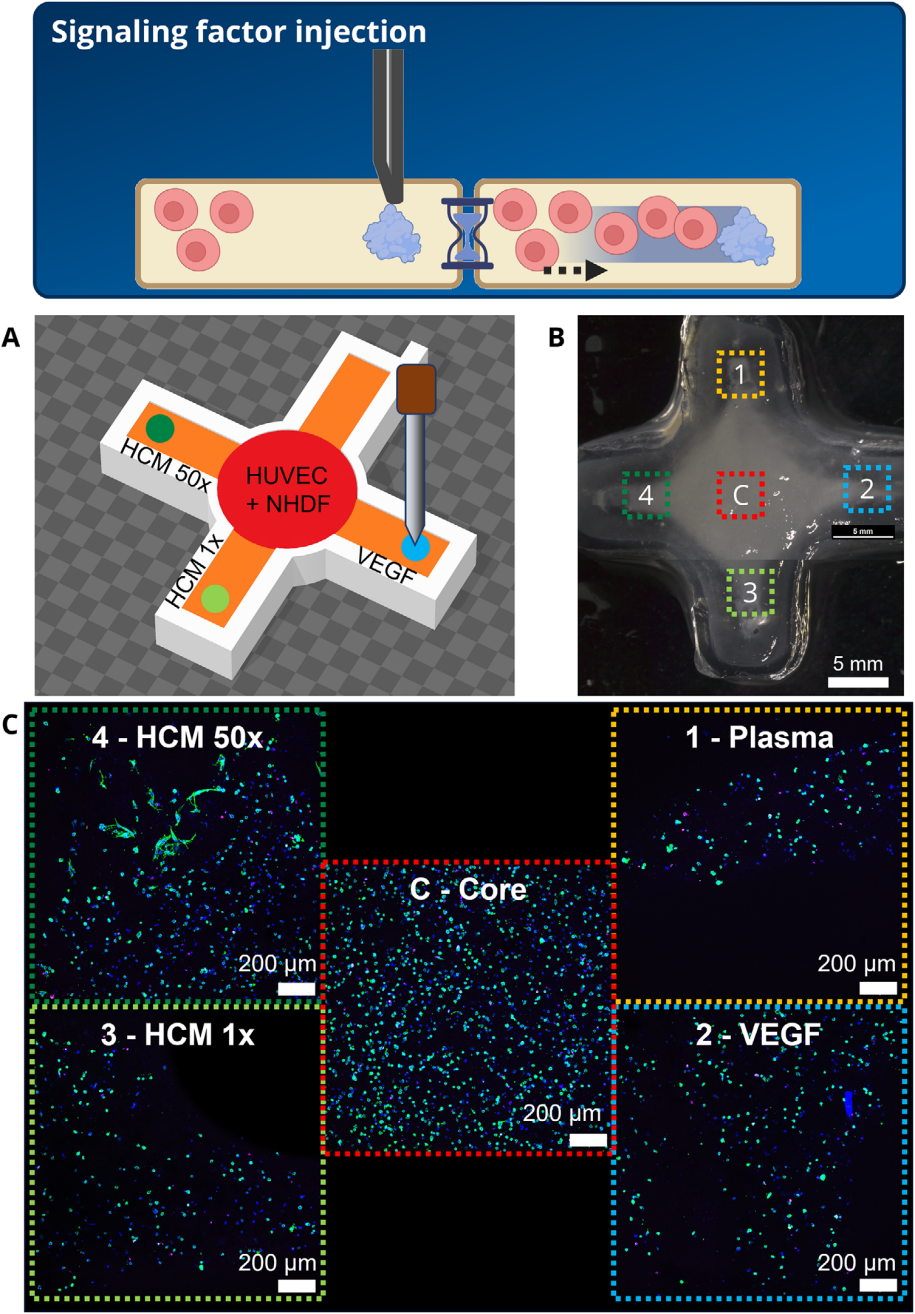
Injection of chemo‐attractive factors into bioprinted constructs to direct cell migration. Injected factors form a concentration gradient; over time, cells migrate in the direction of this gradient. (A) Schematic representation of the printed constructs: A base and a cross‐shaped frame structure were extrusion‐printed using cell‐free AlgMC–water; the central part of this structure (“core”) was extrusion‐printed using AlgMC–plasma loaded with HUVEC (1 × 10^6^ cells/g ink) and NHDF (4 × 10^6^ cells/g ink). The channels in the “arms” (width: 0.84 mm) were filled by DoD printing of cell‐free Alg–plasma. Then, 400 nL of the different signaling factor solutions (VEGF, 1 × HCM, 50 × HCM) were injected in a 4 mm distance to the core, the fourth channel without injected signal factors served as control. (B) Image of the printed and crosslinked construct: AlgMC–water appears transparent while areas containing plasma appear whitish. The sections analyzed by cLSM after 7 days of cultivation are marked. (D) cLSM images of DAPI (blue) and phalloidin (green) stained cells, visualizing cell density in the arms, which were cell‐free before cultivation; three biological samples were analyzed; scale bar: 200 µm.

### 2.4 Bioprinting System

A pneumatic‐driven extrusion printer (Bioscaffolder 3.1; GeSiM, Radeberg, Germany) was used to print AlgMC constructs. Bioinks were extruded through a conic nozzle with 860 µm diameter using 40–50 kPa air pressure. The printing speed was adjusted to achieve uniform strand thickness and varied between 12 and 15 mm/s.

To combine extrusion with DoD printing, the BioScaffolder was equipped with a micro‐pipette as an additional tool on the printing head that can be operated in combination with the extrusion channels. The micro‐pipette consists of a glass needle with a 1 mm diameter and an opening of 130 µm diameter. It is connected to the bioprinter and is operated with sterile water as the system liquid, separated from the sample by an air gap to prevent cross contamination. Samples (maximum volume 10 µL) are aspirated via pumping from a 96‐well reservoir on the printing bed; single drops are dispensed using a magnetic solenoid valve.

### 2.5 Analysis of Cell Viability and Distribution

At different time points of cultivation, bioprinted samples were stained for live and dead cells with Calcein AM/ethidium homodimer 1 (LIVE/DEAD Viability/Cytotoxicity Kit for mammalian cells, Thermo Fisher Scientific, USA) following manufacturer's protocol and imaged with a BZ‐X800 fluorescent light microscope (Keyence Corporation, Japan) in the first experiment or by confocal laser scanning microscopy (cLSM) using a Leica TCS SP5 (Leica Microsystems, Germany) in the second and third experiment. In Experiment 1 (Chapter 3.2), samples were analyzed on Days 1 and 7, samples in Experiment 2 (Chapter 3.3.) were analyzed on Days 2, 7, 14, and 28, and in Experiment 3 (Chapter 3.4), samples were analyzed on Day 7. Semiautomatic area determination of living and dead cells using ImageJ (Fiji, Version 1.52p) [14] was used to assess cell viability which was defined as the ratio of the area of living cells divided by the sum of areas of live and dead cells. At least three images of each sample were taken for the assessment of cell viability.

In the third experiment (described in Chapter 3.4), the samples were washed in PBS, fixed with 4% formaldehyde/PBS for 30 min, permeabilized with 0.1% Triton‐X/PBS for 5 min, incubated in 1% bovine serum albumin (BSA)/PBS overnight and stained with 1 µL/mL DAPI (Gibco Life Technologies) and 25 µL/mL Phalloidin iFluor 488 (Invitrogen, USA) in 1% BSA/PBS for 60 min.

## 3 Results and Discussion

### 3.1 DoD (Bio)Printing With the Solenoid Micro‐Pipette

The micro‐pipette is operated via the bioprinter and collects samples from external sources instead of an internal reservoir or cartridges, setting it apart from most other inkjet printheads. Sample movement with the micro‐pipette is achieved by a system liquid (SL, sterilized water) that is moved by a diluter. The micro‐pipette contains a solenoid‐valve set to an opening frequency of 60 Hz, therefore releasing 60 drops/s. Initial mass flow experiments revealed a single‐drop volume of 30–50 nL, dependent on the viscosity of the sample. Due to the reproducible droplet size and stable pressure, the cell number for each drop can be calculated down to single‐cell placement. To facilitate the printing of samples of different viscosities and maintain similar drop sizes, a pressure compensation vessel is interposed in the tubing: Air pressure, controlled by the bioprinting system, regulates the volume and speed of the moved SL and allows for the printing of liquids in the viscosity range from (water‐like) cell culture medium up to 2% Alg‐based hydrogels. Thus, samples can be either placed onto flat substrate and cultivated without supporting structures if suspended in a hydrogel, or be deposited into an existing construct.

Most DoD bioprinters operate with one of the three techniques: thermal, piezoelectric, or electrostatic inkjet. They differ in the method of how drops are generated, but all can deliver (bio)ink droplets to precise positions on or within the substrate, e.g., a collagen sheet or a hydrogel [3, 15, 16, 17]. All of these techniques use one or more ink‐cartridges to store the sample liquid(s), which greatly increases the necessary amount of sample volume and limits their possibilities to functionalize heterogenous bioprinted tissue models. In contrast, the set‐up of the micro‐pipette utilized in this study allows for the addition of numerous (bio)inks which are collected from a 96‐well reservoir. This enables an easy and more complex functionalization of volumetric bioprinted constructs and the addition of multiple biomolecules and cell types in one process step while only requiring extremely small amounts of sample volume. Thus, this approach prolongs the printing process due to the necessity of frequent sample collection and potential washing steps, yet it is more economical and enables high flexibility. Such an integrated pipetting approach can be down‐scaled to volumes < 1 nL, by using a piezoelectric nanoliter pipette [18].

### 3.2 Combined Extrusion and DoD Printing Allows Local Cell Deposition With a Higher Degree of Freedom

By using the micro‐pipette, cells can be placed not only onto a flat substrate, but also locally defined in a hydrogel construct (Figure 1). As demonstrated in the first experiment for a coculture of NHDF and HUVEC, there are two options for DoD‐based cell deposition into an extrusion‐printed hydrogel construct: (1) by injection of a small volume (five drops) of the bioinks into AlgMC‐plasma strands, exploiting the self‐healing properties of the hydrogel (Figures 1A) and (2) by filling a cavity, such as the channel‐like spaces between AlgMC–plasma strands, with the bioinks by dispensing 25 drops spread out over five spots in 2 mm distance (Figure 1B). In both approaches, the high‐viscosity AlgMC–plasma ink, which enables 3D printing with high shape fidelity [13], forms a mechanical support structure for the DoD bioinks based on fibrinogen solution, whose water‐like viscosity does not allow extrusion‐printing at all, as they simply flow out of the cartridge even without applying pressure [19]. The use of the micro‐pipette in combination with extrusion printing, therefore, opens the possibility of using F‐based bioinks and thus, exploiting its excellent ability to support cell‐matrix interactions and cellular processes such as angiogenesis [20], while compensating for its weak mechanical properties. Even bioinks based on cell culture medium can be precisely placed within a 3D hydrogel construct with this method.

Fluorescence microscopy after live/dead staining indicated high cell viability in both the spot and the channel approach—nearly no dead cells were observed (Figure 1C). Quantification revealed close to 100% viability, which can be attributed to the cell‐friendly properties of fibrin and plasma as well as the comparatively gentle method of DoD bioprinting. On Day 1 of culture, the cells in both approaches started to spread. On Day 7, the cells in the channel approach formed a pronounced network, indicating continuation of cell spreading and proliferation, supported by the pure fibrin matrix. In the spot approach, in which a smaller amount of fibrinogen was injected, which can be assumed to mix with the Alg, the majority of the cells remained at the site of injection, although an increase in cell density was also observed here. Some of the cells started to migrate out of this area, probably supported by the plasma component of the AlgMC hydrogel (Figure 1C). Thus, not only the composition of a bioink and the surrounding hydrogel but also the way in which the bioink is deposited influences the distribution of the cells and ultimately their differentiation into tissue‐like/cellular structures.

### 3.3 Injection of Cells Into Extruded Hydrogel Strands: The Stiffness of the Hydrogel Can Influence the Cell Behavior (Aggregation vs. Migration)

When cells are injected with the micro‐pipette into extrusion‐printed hydrogel strands, the properties of the surrounding hydrogel matrix—physical and (bio)chemical—can influence the cell behavior. This was demonstrated in the second experiment for the parameter “gel stiffness” (Figure 2): hMSGC suspended in the CF ink were injected by DoD printing into bulk constructs, which were generated by 3D extrusion‐printing of either AlgMC–water or AlgMC–PBS (Figure 2A). Both inks are printable with high shape fidelity, but AlgMC–water exhibits a higher viscosity and forms stiffer gels than AlgMC–PBS [11, 12]. During cultivation over 28 days, the cells exhibited in both constructs a high viability, which was close to 100% (Figure 2B). Fluorescence microscopic images revealed in both surroundings spreading of the cells (Day 2) as well as a strong increase in the cell number (Day 7) due to the presence of the cell‐supportive ECM‐proteins collagen type I and fibrin [20, 21]. During further cultivation (until Day 28), the stiffer matrix formed by AlgMC–water induced the formation of dense cell aggregates, whereas the less stiff matrix of AlgMC–PBS allowed the cells to migrate, which resulted in the fusion of two adjacent cell spots (Figure 2C). Thus, while the injection of cells into a softer hydrogel matrix facilitates cell migration and ultimately the formation of larger cellular structures, the injection of cells into a stiffer hydrogel matrix promotes cell aggregation, which could be used for the production of organoids.

### 3.4 Injection of Signaling Factors Into a Bioprinted Structure Can Direct Cell Migration

The micro‐pipette can also be used to place signaling factors at a specific side in a hydrogel construct. Over time, the factors diffuse into the surrounding hydrogel matrix, forming a concentration gradient and thus trigger a cell response. This was demonstrated in the third experiment for chemo‐attractive factors stimulating cells to migrate towards this gradient (Figure 3): A coculture of NHDF and HUVEC suspended in AlgMC–plasma was placed by extrusion‐printing in the center of a cross‐shaped construct; the channels in the “arms” of this construct were filled by DoD printing of cell‐free Alg–plasma and at their ends, the signaling factors were injected (Figure 3A). After cultivation of the crosslinked constructs for 7 days, the presence of cells in the arms was analyzed at defined locations (Figure 3B). Cells were detected in all four arms, indicating cell migration from the cell‐laden core into the cell‐free areas (Figure 3C). The cell density was highest in the arm loaded with 50 × HCM compared to the arms loaded with 1 × HCM or VEGF. This can be attributed to the higher concentration of the factors (50 vs. 1 × HCM) as well as to the fact that HCM contains a mixture of various chemo‐attractive factors that can act synergistically and are therefore more effective than VEGF alone [8, 9]. Cells were also detected in the arm which was not loaded with VEGF or HCM but to a significantly lower amount. This can be explained by the presence of fresh‐frozen plasma as part of the ink, used for filling of the channels. As blood derivative, fresh frozen plasma is rich in proteins containing cell attachment sites, namely fibrinogen and fibronectin, as well as rich in growth factors and cytokines released from the platelets during its fabrication [13, 22]. The observations in this experiment demonstrate that the approach is in principle suitable to direct cell migration within a hydrogel construct. However, a number of influencing parameters such as the type and concentration of the signaling factors, the distance between the signaling factor depot and the cells as well as the properties of the hydrogel matrix through which the cells are to migrate must be adjusted.

### 3.5 Concluding Remarks

The combination of extrusion and DoD bioprinting in one fabrication process allows the precise placement of cells suspended in low‐viscosity bioinks into extrusion‐printed hydrogel constructs. This technique enables the integration of bioinks based on mechanically weak but cell‐supportive biopolymers that are not suitable for extrusion printing alone—such as collagen and fibrinogen—into volumetric 3D constructs. Thus, it easily combines mechanical stability and printing fidelity with a microenvironment that resembles the natural extracellular matrix. When cells were injected into a hydrogel, its stiffness was observed to influence their behavior, with stiff gels leading to cell aggregation, beneficial for organoid formation, while less‐stiff hydrogels tend to support cell migration, which is an important aspect of cell differentiation and organization processes. Further research should investigate other influencing matrix properties, such as biochemical cues, as well as the distance between neighboring cell spots. The injection of signaling factors was demonstrated to direct cell migration, opening up possibilities to control cell organization into larger structures. Besides cell‐attractive factors, also differentiation factors could be applied to conduct developmental processes in such artificial tissue constructs.

In comparison to other DoD printing techniques, the solenoid micro‐pipette setup used herein did not rely on a reservoir connected to the DoD nozzle. Instead, samples were aspirated from external reservoirs. This allows for very small sample volumes, prevents cell sedimentation in the reservoir prior to printing, and enables the addition of various cell types or signaling factors into one construct in a short time span.

## Conflicts of Interest

The authors declare no conflicts of interest.

## Supporting information




**Supporting File 1**: elsc70062‐sup‐0001‐SuppMat.docx

## Data Availability

The data that support the findings of this study are available from the corresponding author upon reasonable request.
