# Supplementary material for: Combination of Extrusion and Drop‐on‐Demand Bioprinting in One Process Enables the Local Placement of Cells or Signaling Factors Into (Bio) Printed Hydrogel Structures
Source: Eng Life Sci. 2025 Dec 30;25(12):e70062. doi: 10.1002/elsc.70062 (PMC12754084; doi:10.1002/elsc.70062)
Supplement: Supplementary file 1 — Supporting File 1: elsc70062‐sup‐0001‐SuppMat.docx [file ELSC-25-e70062-s001.docx]

Supporting information

Technical Report

**Combination of extrusion and drop-on-demand bioprinting in one process enables the local placement of cells or signaling factors into (bio) printed hydrogel structures**

Finn Dani, Nieves Cubo-Matteo, Leonie Schlicht, Michael Gelinsky, Anja Lode

**A**


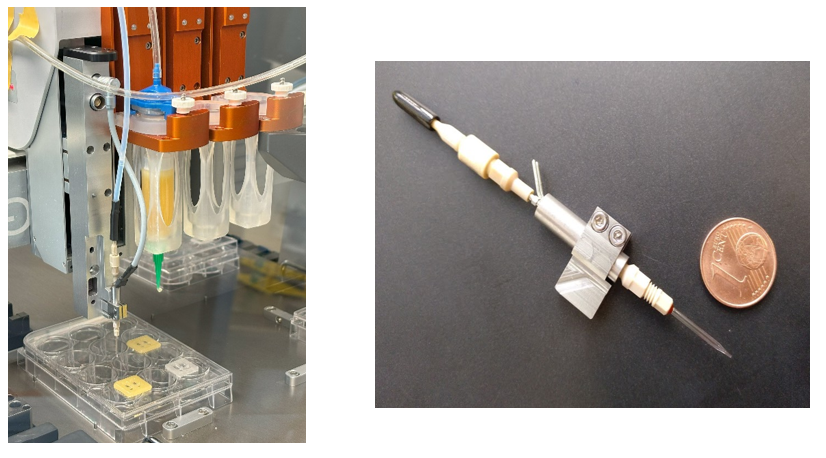


**B**

**Figure S1.** (A) Macroscopic image of the printhead with the solenoid micro-pipette for DoD printing (left) and one cartridge for extrusion printing (right). (B) Close-up of the solenoid micro-pipette.
